# Natural and Artificial Mechanisms of Mitochondrial Genome Elimination

**DOI:** 10.3390/life11020076

**Published:** 2021-01-20

**Authors:** Elvira G. Zakirova, Vladimir V. Muzyka, Ilya O. Mazunin, Konstantin E. Orishchenko

**Affiliations:** 1 Federal Research Center Institute of Cytology and Genetics, Siberian Branch of the Russian Academy of Sciences, 630090 Novosibirsk, Russia; zakirova@bionet.nsc.ru (E.G.Z.); muzyka@bionet.nsc.ru (V.V.M.); 2 Department of Genetic Technologies, Novosibirsk State University, 630090 Novosibirsk, Russia; 3 Skolkovo Institute of Science and Technology, 143026 Skolkovo, Russia; I.Mazunin@skoltech.ru

**Keywords:** mitochondrial DNA segregation, heteroplasmy, selective elimination, mitophagy, mitochondrial engineered nucleases

## Abstract

The generally accepted theory of the genetic drift of mitochondrial alleles during mammalian ontogenesis is based on the presence of a selective bottleneck in the female germline. However, there is a variety of different theories on the pathways of genetic regulation of mitochondrial DNA (mtDNA) dynamics in oogenesis and adult somatic cells. The current review summarizes present knowledge on the natural mechanisms of mitochondrial genome elimination during mammalian development. We also discuss the variety of existing and developing methodologies for artificial manipulation of the mtDNA heteroplasmy level. Understanding of the basics of mtDNA dynamics will shed the light on the pathogenesis and potential therapies of human diseases associated with mitochondrial dysfunction.

## 1. Introduction

Ten years after the discovery of the DNA double helix, Magrit and Sylvan Nass found that mitochondria harbor their own double-stranded DNA (mitochondrial DNA, mtDNA), whose structure differs from the nuclear DNA [1]. Currently, it is established that human circular mtDNA of 16,659 bp in length encodes 37 genes, which are essential for oxidative phosphorylation (OXPHOS) and stable energy production by the cell [2,3].

Highly compact mtDNA possesses many features distinct from the nuclear genome [4], some of which lead to the unique genetic drift of mtDNA. The transmission of the mitochondrial genome is strictly maternal. Uniparental inheritance was first shown in rats [5] and mice [6] and then confirmed in humans [7]. Although several studies debate the biparental mtDNA inheritance [8,9,10,11], such cases seem to be very rare, and their mechanisms remain largely unknown. Secondly, the mitochondrial genome exists in multiple copies. One somatic cell contains around 10^3^–10^4^ molecules of mtDNA [12,13]. Most frequently, a cell has only one type of mitochondrial genome. This condition—homoplasmy—usually occurs when all alleles of mtDNA are clonally expanded. However, considering the increased mutation rate of mtDNA comparing to the nuclear DNA, defective copies of mtDNA could coexist with wild-type alleles in a cell [14,15,16,17]. This phenomenon referred as heteroplasmy is specific to mitochondrial genome.

Different cells in a multicellular organism might have diverse mtDNA species. Moreover, the level of heteroplasmy may vary between the cells of the same tissue or organ, between the organs in one individual, and between individuals in a single family [18]. Such heterogeneity may occur at different developmental stages and proceed in an unpredictable direction. Defining the causes, dynamics, and driving forces of heteroplasmy will provide an insight on the inheritance and the progression of many mitochondrial diseases, the prevalence of which is approximately 1:4300 in the human population [19].

Here, we review several mechanisms of heteroplasmy level regulation and mtDNA segregation in somatic and germ cells, including the natural processes of mtDNA elimination. Additionally, we discuss how cells control the diversity of mtDNA species. Additionally, we review the state-of-the-art approaches (including those under active development) for the artificial modulation of mtDNA heteroplasmy. Finally, we provide specific examples of how these mechanisms can be used for uncovering the pathogenesis and rising novel therapies of mitochondrial dysfunction-associated diseases.

## 2. Mitochondrial Genome Heteroplasmy

It is believed that due to the proximity of the respiratory chain complexes, the absence of histones on mtDNA, and the lack of effective mechanisms of mtDNA repair, the mitochondrial genome is extremely vulnerable to the effects of reactive oxygen species (ROS). However, it is known that mitochondria possess several DNA repair mechanisms present in the nucleus. Most notably, there are base excision repair (BER) and microhomology-mediated end joining (MMEJ). The possibility of the presence of nucleotide excision repair (NER), mismatch repair (MMR), homologous recombination (HR,) and classical non-homologous end joining (NHEJ) is still under extensive debate [20]. In addition to that, even a rescue mechanism for the replication fork has been demonstrated in mitochondria [21]. However, the overall number of effectively proceeding DNA repair mechanisms in mitochondria is somewhat smaller than in the nucleus [20]. Moreover, although it is now known that mtDNA is not naked, the nucleoid structures formed to protect mtDNA are not exactly analogous to histones in the nuclear genome [22], and therefore, mitochondrial DNA is more available for mutagenic agents than the nuclear one. In turn, all this leads to a 10–100-fold increase of mutation rate compared to the nuclear DNA [14,15,16,17]. However, in a recent study, Kauppila et al. [23] demonstrated that the increase of ROS level in mitochondria does not lead to the growth in the number of de novo mutations in the mitochondrial genome. Moreover, point mutations in mtDNA most frequently form as transitions, suggesting the high error rate of mitochondrial DNA polymerase (POLG) during the replication. Based on that, one could infer that the main mutagenesis-inducing factor of mtDNA is an incorrect functioning of mitochondrial DNA replicative machinery. However, we cannot completely exclude the effect of ROS, intermolecular recombination, and specific mechanisms of mtDNA repair in mtDNA mutation formation.

MtDNA mutations could lead to the manifestation of a wide spectrum of neuromuscular and neurodegenerative diseases [24]. Regardless of the cause, the mutated copies of mtDNA can create the heteroplasmy within the cell. Depending on the mutation, cell, and tissue type, the critical heteroplasmy level for the manifestation of mitochondrial disease varies between 60% and 90% of defective copies [25]. The differential sensitivity of organs to physiological changes in mitochondria is a crucial parameter to determine the heteroplasmy threshold effect. The threshold in highly aerobic tissues such as muscle, heart, and central nervous system is usually lower than in less aerobically active tissues [26]. In addition to the tissue specificity of the heteroplasmy threshold level, it also depends on whether it is a point mutation (average critical level >80%) or a deletion (>60%) [27,28].

Until recently, it was considered that mtDNA heteroplasmy is an extremely rare event and that its threshold level is achieved due to a clonal expansion of mutant mtDNA molecules during the development [29]. However, ultra-deep resequencing revealed that the majority of human population, regardless of age, has low-level heteroplasmy in many tissues [30,31]. The universality and the prevalence of heteroplasmy cannot be explained only by spontaneous mutagenesis during early development. A portion of heterogenous mtDNA is inherited, which is proved by the presence of some of the low-frequency mutant alleles in both offspring and mothers [32]. Once emerged, somatic and inherited mutations might either reach a threshold level during life or be transmitted to the next generation preserving the low abundance, or even be completely eliminated after a certain time [33]. Therefore, the heteroplasmy is dynamic, and the behavior of mtDNA haplotypes segregation is rather unpredictable.

For the first time, a heteroplasmic shift within one generation was reported for the Holstein cows [34]. It is well established that one of the critical events affecting the genetic drift of mtDNA is the female germline bottleneck during the oogenesis. Resulting from it, the number of mtDNA copies decreases from 100,000 in the fertilized oocyte to 200 in a primordial germline cell [35,36,37,38]. Therefore, only a selected subpopulation of mtDNA would become prevalent in the formation of the mitochondrial bioenergetics function in the next generation.

Currently, a bottleneck theory is confirmed in multiple model organisms and systems, including early embryogenesis in humans [16,37]. Mathematical and statistical models demonstrated that the decrease in the amount of mtDNA in the germline cells is sufficient to shift the heteroplasmy level considerably between mother and offspring [39,40,41,42]. Importantly, a bottleneck theory predicts only a stochastic mtDNA segregation during embryogenesis, without considering a potentially significant change in heteroplasmy level during the entire span of individual development. The rate of the genetic drift of mtDNA might be affected by the vegetative segregation of mitotic cells, the preferential replication of certain mtDNA subpopulations, the subcompartmentalization of mtDNA into separate homoplasmic clusters, and a number of other less studied factors [18,36,43]. Notably, ROS have been shown to direct mtDNA segregation toward homoplasmy in human primary fibroblasts via a linear concatemer formation during mtDNA replication [44]. In addition, the intracellular mitochondrial quality control system might affect the consequent clonal expansion of certain mtDNA variants. Although it is currently impossible to determine which of the above mechanisms is the most relevant, it is obvious that together, they can lead to changes in the intracellular level of heteroplasmy during development, and, possibly, to the manifestation of mitochondrial disease.

## 3. Natural Mechanisms of mtDNA Elimination

### 3.1. A Selective Elimination of mtDNA in the Germline

Previously, it was considered that the animal mitochondrial genome undergoes neither positive nor negative selection during the evolution. However, after analyzing the inheritance of mtDNA in different model organisms, several groups established that mtDNA segregation proceeds under an intense negative selection during individual development [39,45,46]. After assessing the dependence of heteroplasmy level in the offspring on the mother’s heteroplasmy level for several mutations, Stewart et al. demonstrated that pathological mutations are selectively eliminated during female oocyte maturation in mice [47]. Following this work, there have been multiple papers focused on the effects of certain mutations on mtDNA segregation in fruit fly [46,48], mouse [49], and human [16]. In case of the presence of two different mitochondrial mutations within mouse mitochondrial genome, a directional selection proceeded against a severe mutation causing an open reading frameshift in the *ND6* gene. At the same time, a milder missense mutation in *cytochrome c oxidase I* (*CoI*) gene was retained despite causing myopathy and cardiomyopathy in mice [49]. These results suggest the presence of effective complementation in the mitochondrial genome, leading to the elimination of mutations with a strong pathogenic effect while maintaining less pathogenic ones [46]. The mutation type and the possibility of its inheritance also correlate with the mutation localization in the mitochondrial genome. For instance, mtDNA variants with mutations in the D-loop are transmitted more frequently than the variants with mutations in ribosomal RNA genes, while mtDNA mutations of structural and transport RNA genes are transmitted to the next generation with similar frequencies [16]. As for protein-coding genes, mtDNA molecules with synonymous mutations are more frequently transferred to the next generation than nonsynonymous [47,50]. Overall, the above studies suggest that the negative selection in female germline cells helps to maintain and expand mtDNA variants that do not decrease the activity of the mitochondrial respiratory chain.

#### Potential Mechanisms of mtDNA Negative Selection

There is scarce knowledge of the negative mtDNA selection during female germline development. One study suggests that it proceeds before the germline bottleneck [51]. By eliminating oocytes with pathological mtDNA mutations, negative selection decreases the probability of a severe mitochondrial disease manifestation and promotes non-pathological mtDNA haplotypes to the next generation [52]. The mitochondrial quality control (MQC) process is considered to predominantly regulate mtDNA variant selection [16]. PTEN-induced kinase 1 (PINK1)/Parkin-mediated mitophagy is the most well-established way of MQC [53]. It is initiated in response to the increase of the proportion of defective components of the OXPHOS system, leading to selective elimination of the affected organelles with pathogenic mtDNA molecules [54]. In response to the mitochondrial dysfunction, PINK1 kinase stabilizes at the outer mitochondrial membrane (OMM) and consequently recruits ubiquitin E3 ligase Parkin [55,56] and several other autophagy receptors to the OMM [57]. Recruited enzymes ubiquitinate mitofusins (MFNs) and other proteins located at the OMM and are responsible for its fusion [58]. In turn, this inhibits the fusion of defective mitochondria to a single reticular network [59]. The suppression of mitochondrial fusion in heteroplasmic cells could lead to the physical isolation of mitochondria containing mutant mtDNA for preventing the functional complementation with wild-type molecules of mtDNA. In human cybrid cells derived from multiple different tissues and containing the *CoI* gene mutation, it has been shown that *Parkin* overexpression could induce mitophagy [53]. The overexpression of *Pink1* and *Parkin* also leads to a selective elimination of mutant mtDNA in muscles of adult *D. Melanogaster* [60]. In contrast, *Parkin* knockout in *C. elegans* cells containing 60% of mtDNA with large-scale deletion [61] leads to a heteroplasmy shift toward mutant mtDNA [62].

At first glance, negative selection via mitophagy matches well with a bottleneck hypothesis explaining the mtDNA amount reduction during the oogenesis. However, several researchers suggested that a bottleneck might form not only due to a reduction in the number of mtDNA molecules but also due to a focal replication of mtDNA subpopulation during oogenesis [51,63] and via the packaging of several mtDNA molecules into segregation units [36,64]. Both pathways visibly limit the effective size of the mtDNA population, leading to intensified segregation of the selected mtDNA pool and consequent heteroplasmic shift in developing oocytes [65].

To test the hypothesis of the elimination of defective mtDNA copies via selective wild-type mtDNA replication, Hill et al. analyzed mtDNA selection in early embryonic oogenesis in multiple generations of *D. melanogaster* [48]. Fruit flies with homoplasmic temperature-sensitive mutation *mt:CoIT300I* in *the cytochrome c oxidase* (*CoI*) gene have an impaired eclosion phase and early lethality (within 5 days) during the incubation at increased temperature (29 °C). Heteroplasmic flies are not killed at high temperature. Nevertheless, among the offspring, flies with wild-type mtDNA were predominant. The identified dependence of the fly development on the incubation temperature enabled determining how the degradation of the high temperature-sensitive mtDNA variants proceed at the molecular level. It was shown that mtDNA selection starts as soon as the replication in the late germarium. *mt:CoIT300I* mutation causes a reduction of cytochrome C oxidase activity at high temperature, consequently blocking mutant mtDNA replication in the germ cells. However, how is oxidative chain impairment associated with mtDNA replicative activity? This phenomenon has been explained by Stewart et al. [18]. It is established that all mitochondrial replicative proteins are encoded by nuclear genes and are imported from the cytoplasm. The transfer of these proteins depends on the mitochondrial membrane potential reflecting the functional state of mitochondria [66]. As a result, intermembrane protein transport is suppressed in response to the decrease of the membrane potential and of the electron transport chain (ETC) activity. In turn, this leads to the blockage of synthetic processes in defective organelles. Therefore, the formation of the germline cells of the next generation is under the nuclear genome control, and thus positive or negative mtDNA selection happens depending on the synergy between the nuclear and the mitochondrial genome. The driving forces of the negative selection are reviewed below.

According to the segregation units model, the bottleneck in the female germ cells proceeds without a reduction in the mtDNA content. It potentially takes place due to the packaging of mtDNA molecules in a small number of homoplasmic clusters, which segregate into daughter cells during mitosis [36,64]. Jenuth et al. have identified around 200 segregation units in mice [37]. This number resembles an absolute quantity of mtDNA molecules in forming mouse oocytes [40]. Considering the fact that on average, one nucleoid carries approximately 1.4 mtDNA molecules [67], one could assume that the nucleoid is the unit of a homoplasmic clustering and determines the bottleneck width. This parameter defines which mtDNA molecules would shape the functional characteristics of future oocytes.

The negative selection mechanisms presented above contribute to a decrease in the number of mtDNA molecules before or during the bottleneck. Moreover, these processes take place at the mitochondrial level. Meanwhile, mtDNA degeneration can occur at the cellular level. For example, primary oocytes with a high load of mtDNA mutations can also be selectively degraded (follicular atresia) [68].

Currently, there is no direct empirical evidence as to which selection mechanism is key in the germline bottleneck. Moreover, the functioning of these processes in somatic cells (for example, mitophagy) is not excluded. In any case, they all modulate the natural drift of mtDNA.

### 3.2. Dynamics of Heteroplasmy in Somatic Cells

Unlike in the germline, mtDNA mutations in somatic cells frequently demonstrate neutral patterns of segregation [69]. Recently, investigators have described several cases of both positive and negative somatic selection of mutant mtDNA subpopulations [70,71,72].

For instance, a gradual reduction in the number of mtDNA molecules with mt3243A>G mutation in a gene encoding *tRNALeu(UUR)* in the patient’s hematopoietic stem cells has been reported [73]. For example, the reason for such an exponential decrease of mutant mtDNA content could be spontaneous mtDNA vegetative segregation between daughter cells or a directed selection against the pathogenic mutation [70]. One of the pathways of directed selection might potentially be through the fragmentation of the mitochondrial network and the elimination of impaired mitochondria by mitophagy. It becomes possible if the mutation severely affects cell function and viability. A similar reduction of the copy number of mtDNA with mt3243A>G mutation has been detected in epithelial [71] and buccal [74] cells during aging. The strong correlation between the age and the heteroplasmy level matches well with the presence of a negative selection in mitotic cells. The mt3243A>G mutation mentioned above for the dividing cells segregates differently in neuronal model in vitro. Surprisingly, compared to hematopoietic stem and epithelial cells, iPSC populations differentiated into neurons in vitro demonstrated stable heteroplasmy levels upon both serial passaging and differentiation [75]. Moreover, iPSC-derived neurons retain not only the heteroplasmy level but also the parental cell pathological phenotype [76]. Overall, mtDNA segregation process largely depends on the examined cell type revealing the oxidative chain performance as the major factor for such heterogeneity [77].

MtDNA segregation is a continuing process happening not only in developing gonads and in dividing cells but also in many post-mitotic tissues throughout the lifespan. Inherited at low frequency or acquired during the life, pathogenic mtDNA variants could reach a biochemical threshold level at any age due to the relaxed replication of mtDNA [78]. Works on *C. briggsae* [79] or human cybrid cells [72], containing mitochondria with partially deleted mtDNA, demonstrated the preferential accumulation of defective mtDNA molecules. The segregation toward mutant variants could be associated with reduced mtDNA size following a deletion because the repopulation of shorter molecules is much faster than of full-size wild-type mtDNA. Despite potential negative effects of mutations on mitochondrial function, a similar mechanism may be also inherent for the germline cells.

An unexpected positive effect of mtDNA negative selection failure was found in the oncogenesis of several cancers. Heteroplasmic mtDNA mutations affecting *ND1-6* and *ND4L* genes stimulate tumor growth by activating anti-apoptotic pathways [80,81,82,83]. At the same time, reaching a certain threshold heteroplasmy level or mutant mtDNA homoplasmy promotes the inhibition of cancer cell mitochondrial function and the tumor suppression [84,85,86]. In turn, this leads to a better recovery rate for patients with breast cancer or acute myeloid leukemia [87,88]. The negative correlation between mtDNA mutation burden and the tumor progression suggests the existence of a compensatory mechanism for cancer cell elimination and organism protection against metastasis and disease relapse. However, the practical application of directed mtDNA mutagenesis is limited due to the lack of the information on the formation and development of different tumor types.

**Figure 1:**
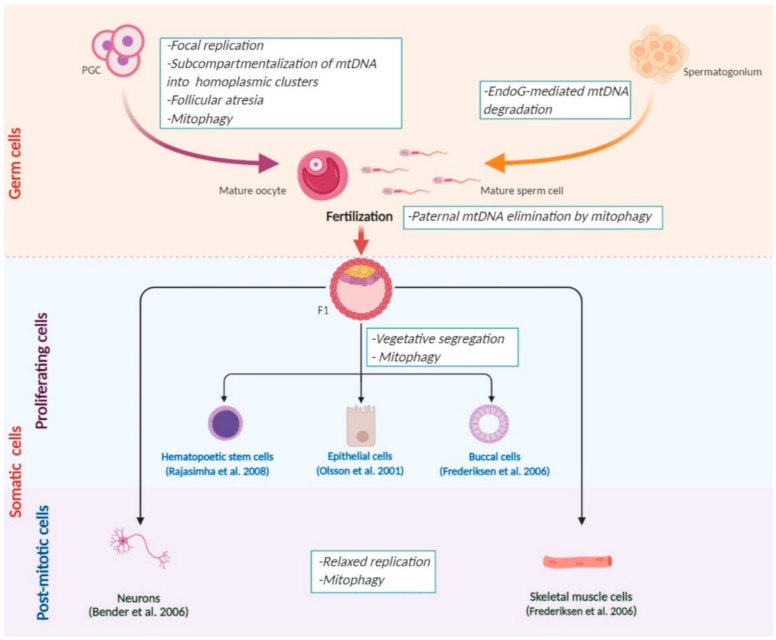
The genetic dynamics of mitochondrial DNA (mtDNA) is regulated via diverse mechanisms during mammalian development. In the maturation process, female oocytes undergo negative selection supposedly happening just before the developmental bottleneck. The oocytes with high mtDNA mutation load could be eliminated via follicular atresia. At the same time, during spermatogenesis, endonuclease G (EndoG)-mediated mtDNA degradation occurs. During fertilization, the mitophagy of male mitochondria takes place to prevent paternal mtDNA transmission to the next generation (F1). Since the early stages of embryogenesis, mtDNA is randomly distributed between daughter cells during mitosis due to vegetative segregation. In non-dividing cells, one of mtDNA variants might have a replicative advantage due to relaxed replication. These two mechanisms might proceed in parallel in different somatic tissues within one organism to regulate the genetic dynamics of mtDNA. Additionally, mtDNA heteroplasmy can shift toward healthy or mutant variant in development because of the mitophagy process (figure created in BioRender.com).

All of the mechanisms that affect mtDNA segregation during development are summarized in Figure 1.

However, currently, we lack unambiguous answers to the following questions: how does directed mtDNA selection go; how many bottlenecks do cells undergo during the oogenesis; what is the dynamics of segregation for different mtDNA mutations? It is obvious that identified factors, which affect mtDNA dynamics, are not mutually exclusive and represent a part of a unified process, which requires further extensive investigation.

### 3.3. Paternal mtDNA Degradation

As mentioned earlier, mtDNA is inherited maternally in the majority of animal species. The meaning of this inheritance remain poorly explored. Previously, it was hypothesized that the difference between male and female gamete sizes could block the compatibility of paternal and maternal mitochondrial genomes in a zygote [89]. However, a unicellular algae *C. reinhardtii* has similar in size gametes of two sexes, but still, only the maternal mitochondrial genome is inherited [90]. Considering the “unified model of organelle inheritance”, the growing evidence is accumulating for uniparental transmission of mtDNA to prevent the spread of selfish (fast replicating) alleles in the population [91,92]. Such elements can be mutant sperm genomes that replicate faster than the wild-type genome but do not adapt to the egg nucleus [93]. In addition to that, a possible reason for the maternal inheritance of mtDNA is a tendency of cells to maintain more metabolically effective variants in the homoplasmic state [77,94]. However, uniparental inheritance is evolutionarily unstable, because mitochondria are subject to Muller’s ratchet [93].

To support a strictly maternal inheritance and to decrease the competition between parental mtDNA species, several mechanisms of paternal mtDNA degradation in both spermatogenesis and fertilization evolved during the evolution.

#### 3.3.1. MtDNA Elimination during Spermatogenesis

DeLuca and O’Farrell analyzed all stages of male germ cells development of *D. Melanogaster* to track mtDNA fate during spermatogenesis [95]. Spermatids of Drosophila undergo a physical transformation during maturation. At the sperm individualization stage, spermatids get rid of almost all cytoplasm with the majority of organelles, including mitochondria. Mature spermatozoon still contains the remaining mitochondria but without mtDNA. A two-step mechanism is responsible for mitochondrial genome elimination. It includes primary nucleoid degradation with Endo G [95,96] combined with the elimination of remaining aggregates via their transport into caudal “waste bags”, which are then extruded out of the spermatozoon [97]. In addition to Endo G, the molecular machinery to remove mtDNA in fruit fly spermatozoon contains a α-subunit of mitochondrial polymerase γ (Pol γ). Supposedly, the physical proximity of proteins responsible for the replication and the degradation of mtDNA underlies a balanced and stable nuclear control of mitochondrial genome copy number [98]. However, this hypothesis still needs further experimental confirmation.

Do the mammalian species have similar mechanisms of mtDNA degradation before the fertilization compared to Drosophila? Mouse sperm exhibits a three-fold decrease of mtDNA quantity during its maturation, leading to the impaired fertility in mice [99]. A reverse effect associated with the increase of mtDNA level in sperm is found in human males with oligozoospermia and asthenozoospermia [100,101,102]. Several studies suggest that the principal mechanism of mammalian mtDNA degradation is the poly-ubiquitination of defective mitochondria and its consequent proteolysis in the epididymis duct [103,104]. In mammals, including humans, mitochondria ubiquitination in sperm was confirmed by the co-localization of the ubiquitin labeling with mitochondria at all stages of spermatogenesis from spermatogenic cell to mature spermatozoon in fertilization [105,106].

#### 3.3.2. Paternal mtDNA Elimination during Fertilization

A striking example of purifying selection is the elimination of mtDNA from the spermatozoon introduced to the oocyte during fertilization. It was initially considered that sperm mtDNA is not detected in the offspring due to a substantial dilution in the large amount of the oocyte mtDNA [8,97,107]. However, such model of a “passive dilution” has not been confirmed extensively due to the lack of a unified opinion on the number of mtDNA molecules in mammalian spermatozoon [108,109,110]. Studies in this field allowed formulating a hypothesis on the mechanism of a directed degradation (“active elimination”) of paternal mtDNA in a zygote [111,112]. A convincing evidence from a time-lapse fluorescent microscopy from different stages of embryogenesis of *C. elegans* has demonstrated that sperm mitochondria easily get into the oocyte; however, two hours after fertilization, they are all eliminated via autophagy [113,114]. Paternal mitochondria become depolarized in the oocyte. Its inner mitochondrial membrane (IMM) permeabilizes, which allows Endo G transfer from the intermembrane space of male mitochondria into the mitochondrial matrix [97,115]. This leads to the cleavage of spermatozoon’s mitochondrial DNA [116]. MtDNA degradation promotes the formation of autophagy initiation and proteasome degradation signals on the surface of mitochondria [104,117,118]. When the assembly of the autophagolysosome is inhibited, paternal mtDNA molecules are retained until the late embryonic stages and contribute to the formation of bioenergetics function of mitochondria in the next generation. Additionally, the co-localization of autophagy markers (LC3, GABARAP, and p62) with paternal mitochondria has been detected in zygotes of mice [113,119]. Most likely, mitophagy proceeds via the Parkin/Mitochondrial ubiquitin ligase 1 (MulI)-dependent pathway [120]. Despite the differences in the pathways of mitophagy activation caused by fertilization, it is safe to assume that the elimination of mtDNA via autophagy is a conserved mechanism among the absolute majority of animal species [121]. Sato et al. and Kaneda et al. review the information on the paternal mtDNA degradation in different animals in detail [121,122].

Despite the growing evidence of active elimination of paternal mtDNA in mammals, any accurate information about its fate in fertilization and embryo formation is still lacking [112]. However, there are already several well-documented cases of paternal mtDNA inheritance in human and the scientific community aims to explore such events. [10,123]. It is highly possible that a false impression of paternal mtDNA inheritance could be formed due to the presence of NUMT (“Nuclear copies of mitochondrial genes”) pseudogenes, which are integrated into nuclear genome due to intergenomic recombination. However, there are no direct experiments proving this as for now; thus, one cannot exclude rare biparental mtDNA transmission events.

### 3.4. The Driving Forces of mtDNA Segregation

More than 20 years ago, Jenuth et al. have identified the competition between mtDNA haplogroups during the transfer from mother to offspring [37]. However, the molecular basis of this phenomenon has stayed unexplored for a long time. To reveal the factors influencing mtDNA segregation, special conplastic mice were produced [124,125]. Heteroplasmy preferentially shifted toward the C57 variant in the germline of conplastic mice possessing the C57 mouse line nuclear genome and different (C57 and NZB) mtDNA haplotypes [126]. Such a preference could be explained by the functional complementation of the nuclear and the mitochondrial genomes during OXPHOS system assembly. In mitochondria, around 70 proteins for the OXPHOS system encoded by the nuclear DNA and 13 structural proteins encoded by the mtDNA have to physically complement each other to form functional multiprotein respiratory complexes. The incompatibility between the products of the nuclear and the mitochondrial genomes promotes the reduction of mitochondrial activity and cellular adaptive reaction at the early embryonic stages [125]. That makes the heteroplasmic state unstable and promotes a selective segregation of cognate (to the respective nuclear DNA) mtDNA haplotype in the germline [94].

To understand the mito-nuclear heteroplasmy regulation, it is crucial to know the components of the retrograde and the anterograde signaling maintaining certain mtDNA haplotypes. After analyzing mtDNA segregation during the lifespan in 19 different tissues of heteroplasmic C57/NZB mice with C57 nuclear background, it was determined that there is an association between cellular metabolic program, oxidative phosphorylation, and mitochondrial genome segregation [77]. It is known that the ETC of any cell type is tuned to use of certain molecules as energetic substrates and, therefore, for a certain metabolic pathway (for instance, glucose or lipid metabolism). In the case of mtDNA homoplasmy, the function of the respiratory chain is not altered compared to the default metabolic program. In the case of heteroplasmy, there is a dose-dependent imbalance between cellular metabolism and the ETC activity, which in turn induces the respiratory chain rearrangement and intensive ROS production [127]. In response to the increase of ROS level, the proteins encoded by the nuclear genome SCAF1 (supercomplex assembly factor 1), NNT (mitochondrial NAD(P) transhydrogenase), and OMA1 (metalloendopeptidase, which is responsible for the control of the mitochondrial fusion) are imported into mitochondria to fine-tune the oxidative phosphorylation and to reduce the ROS level [128]. At the same time, the ETC dysfunction activates factors that coordinate the replication of mtDNA, mitochondria biogenesis, and mitophagy [129]. In the end, a suboptimal mitochondrial function due to heteroplasmy leads to the degradation of mitochondria, which have mtDNA variants generating excessive ROS.

Systematic studies of the driving forces of mtDNA heteroplasmy have considerably broadened the understanding of the mechanisms of mtDNA segregation and the interaction between the nuclear and the mitochondrial genomes. During the parallel evolution of these genomes, the pathways to coordinate a stable mitochondrial function were established. Along those lines, mtDNA segregation and elimination potentially maintain the mitochondrial homoplasmic state. Consequently, homoplasmy is more preferable than heteroplasmy, because it provides more stable energy input for the cell and reduces ROS production. The alteration of any component of this mechanism would potentially lead the cell, the tissue, or the organism to death.

## 4. Artificial Mechanisms of Mitochondrial Genome Elimination

Mitochondrial pathologies progressing with age do not currently have any common term and are classified into different subgroups of human diseases according to the International classification of human diseases (ICD-11) of the World Health Organization. The lack of a shared etiology and pathogenesis of mitochondrial diseases causes complications in patient’s diagnostics and therapy. Modern clinical diagnostics of mitochondrial pathologies with traditional histochemical, immunohistochemical, and biochemical techniques in combination with high-throughput screening of mitochondrial and nuclear DNA considerably improves early diagnostics [112]. However, in many cases, after determining a type of mitochondrial pathology, a clinician is not able to find an adequate curing approach due to the lack of such disease-suppressing therapies in clinical practice. Sometimes, patients undergo neurotrophic and metabolic therapies aimed at temporal symptomatic suppression [112,113,114,115]. At the same time, modern reproductive and gene therapy techniques not only enable reducing the heteroplasmy level in a patient’s somatic cells but also prevent a transfer of pathogenic mtDNA copies to the next generations.

### 4.1. Reproductive Technologies for the Prevention of Mutant mtDNA Transfer

Women with high heteroplasmy levels in oocytes are often diagnosed with infertility, because the reduced activity of the OXPHOS complexes leads to the suppression of embryonic development at early stages [63,130]. In vitro fertilization (IVF) with preimplantation genetic diagnostics (PGD) of embryos of mtDNA mutation load has become an effective instrument of clinical practice for mitochondrial disease prevention. During this procedure, one or two cells from embryos are taken for the analysis of heteroplasmy level to assess the risk of mitochondrial disease manifestation. For a birth of a healthy child, it is crucial to have a low (<5%) heteroplasmy level in the embryo [131]. It is assumed that this level is similar in all the cells and stable during the course of embryonic development. Considering the fact that mtDNA segregation mechanisms during embryogenesis are not fully studied, one cannot exclude the return to the initial heteroplasmy level in the whole embryo or in its specific tissues. In addition to that, some women have a significant number of mutant mtDNA molecules in the majority of oocytes; therefore, PGD does not assist well in such cases [132]. Instead, to prevent the transfer of mutant mtDNA to the offspring, mitochondrial replacement therapy (MRT) or mitochondrial donation techniques were established [133,134,135]. This technology was first tested on animals [136,137,138]. Thereafter, it has been used in humans to prevent the transmission of mitochondrial diseases. The procedure might include the transfer of a maternal spindle (MST) [138,139], of a pronucleus (PNT) [140], or of a first polar body [141,142,143,144] into enucleated oocytes or zygotes of a healthy donor. As a result, a child born with MRT would have nuclear DNA from both parents but mitochondria from a donor woman. However, despite the reports about successful births of children from “three parents” [140,145], the extensive application of mitochondrial donation still remains impractical or even illegal in many countries due to unresolved technical and ethical issues [146,147,148].

The reversal of embryos to a pathogenic mitochondrial type represents one of the encountered technical problems. It is considered to occur with low possibility because during the MRT, less than 3% of maternal mtDNA happen to be transferred [132,138]. However, it was shown that around 15% of embryonic stem cells produced from the embryos after MRT show a complete return to the initial, mutant mtDNA variant [149,150,151,152]. Such a phenomenon might be based on a reciprocal interaction between the nuclear and the mitochondrial genome [153]. Another crucial disadvantage of mitochondrial donation is that it not only reduces the level of heteroplasmy in the next generation but also does not block the transfer of mutant mtDNA completely. This issue could be resolved using the combination of mitochondrial donation with gene therapy tools [154,155].

### 4.2. Gene Therapeutic Approaches for the Prevention of Pathogenic mtDNA Transmission

In addition to the previously discussed mitochondrial genome characteristics, another critical trait is the retention of mtDNA copy number in a cell. MtDNA haplotypes could degrade due to multiple reasons leading to the overall reduction of mtDNA pool in mitochondria. Surpassing a critical threshold of mtDNA content could cause the death of not only mitochondria but the whole cell. To avoid cell death, mtDNA haplotypes unaffected by degradation factors actively replicate to restore the initial mtDNA copy number [156]. If one could direct natural elimination mechanisms against pathogenic mtDNA molecules, it is possible to repopulate the mitochondrial genome with wild-type molecules to restore the impaired mitochondrial function. Many actively developed gene therapeutic approaches for shifting heteroplasmy level are based on the principle mentioned above. These therapeutic instruments are being used in oocytes to prevent pathogenic mtDNA transmission to the next generation, as well as in somatic cells to treat mitochondrial diseases [154].

#### 4.2.1. Anti-Replicative Approaches

One of several possible ways to shift heteroplasmy toward the wild-type variant is to suppress specifically the replication of mutant mtDNA. For the first time, this methodology was demonstrated using peptide nucleic acids (PNAs) [157]. PNAs are the artificial analogs of oligonucleotides, in which a pseudo-peptide scaffold replaces the sugar phosphate one. The latter one contains N-(2-aminoethyl) glycine as a monomer. The PNA structure makes it more resistant to nuclease and protease cleavage [158] and enables introducing modifications to directly import PNAs into the mitochondrial matrix [159,160]. Additionally, PNAs bind to the complementary single-stranded DNA with higher affinity than analogous complementary DNA [161]. PNA:DNA duplexes with single nucleotide mismatches are more stable than similar DNA:DNA duplexes [162]. Using in vitro replication in physiological conditions, Taylor et al. demonstrated that PNAs might selectively inhibit the replication of the respective single-stranded mtDNAs with deletions or single-nucleotide mutations [157]. Nevertheless, no striking results have been demonstrated on either cell culture or isolated mitochondria. Other than the rather effective import of PNAs into the mitochondrial matrix, the researchers did not observe any inhibition of mutant mtDNA replication [159,160]. That could be explained by the ineffectiveness or complete absence of PNA binding to mtDNA or by the displacement of PNA molecules away from the mtDNA during its replication or transcription. In any case, currently, PNAs could not be used for heteroplasmy shifting, and further experiments are necessary to search for potential PNA modifications to improve the efficiency of target DNA binding in living cells.

Another group has been developing a similar approach to shift the heteroplasmy level. For the selective inhibition of mutant mtDNA replication, they used short RNA molecules with a region complementary to a target mtDNA sequence. Additionally, they inserted F- and D-stem loops from yeast transport RNA (tRNALys(CUU)) or the α and γ domains of 5S human ribosomal RNA into the structure of their recombinant anti-replicative RNAs. As demonstrated earlier, these fragments could promote effective RNA import inside mitochondria [163,164,165]. The supposed mechanism of action of anti-replicative RNA molecules is associated with the incapability of helicase Twinkle in mitochondrial replisome to displace RNA in short RNA–DNA duplexes [166]. Using cultured cybrid cells, it was demonstrated that the anti-replicative RNA approach causes heteroplasmy level reduction for large deletion associated with Kearns–Sayer syndrome and for pathogenic point mutation A13514G in the *ND5* gene. Notably, only some of the tested RNA variants induced the suppression of mutant mtDNA replication [167,168,169]. Similar to PNA methodology, it is questionable whether a single-stranded mtDNA is available for the anti-replicative RNA during the replication and the effectiveness of their binding. Additionally, although many studies demonstrate the transport of different RNAs inside mitochondria (detailed review by [170]), currently, there are no common opinion on the molecular mechanisms of nucleic acid trafficking inside mitochondria and its possible function there [171].

#### 4.2.2. Anti-Genomic Approaches

An alternative method for the reduction of heteroplasmy level toward wild-type mtDNA is a specific elimination of pathogenic mtDNA. Considering the nature of mitochondrial genome multicopy, mechanisms of mtDNA repopulation, and the apparent however debated inefficiency of active mechanisms of double-stranded break (DSB) repair of mtDNA [20,172], one could speculate about the high effectiveness of a directed DSB introduction into mutant mtDNA. Several studies illustrate the presence of some DSB repair mechanisms taking place in the mitochondrial genome—namely, homologous recombination (HR) and non-homologous end joining (NHEJ). One of the first indicators of HR in mitochondria was the finding of circular dimers and catenanes in mammalian mtDNA [173,174,175,176]. Moreover, human heart cells possess a unique configuration of complex mtDNA catenated networks [177]. Another evidence favoring the possibility of the HR activity in mammalian mitochondrial genome is the identification of maternal–paternal mtDNA hybrids in muscle cells of a unique patient [178]. This is an unprecedented case for humans, although the recombination of mtDNA variants from both parents has been detected in other vertebrate and non-vertebrate animal species [179,180,181]. Thirdly, studies on the artificial introduction of DSBs into the mouse mitochondrial genome have shown the occurrence of interspecific DNA exchange between different mtDNA haplotypes [182,183]. All this, plus the identification of several analogs of well-identified in yeast mtDNA HR protein participants in mammalian mitochondria suggest that HR is possible in mammalian mtDNA even in physiological conditions [184]. Another potential mechanism of DSB repair in the mitochondrial genome is one particular type of NHEJ—microhomology-mediated end joining (MMEJ), as opposed to classical NHEJ [185]. It was indirectly confirmed when Meiotic recombination 11 (Mre11) and Poly(ADP-Ribose) Polymerase 1 (PARP1)proteins were found to localize in mitochondria and interact with mtDNA [186,187], and then, it was directly demonstrated to be the principal DSB repair process in mitochondria [185].

Nevertheless, after a DSB is introduced, most of the linear mtDNA molecules rapidly degrade due to the activity of mitochondrial replicative proteins (mtDNA polymerase γ—POLG, DNA helicase Twinkle, exonuclease MGME1) [188], leading to heteroplasmy shifts. For DSB presentation into DNA structure, different nucleases could be used. The majority of proteins essential for mitochondria functioning are encoded in the nuclear DNA. Therefore, TIM (trafficking through inner membrane)/TOM (trafficking through outer membrane) machinery has been formed for the protein transport through the inner and the outer mitochondrial membranes, respectively. The presence of a Mitochondrial Targeting Sequence (MTS) at the protein N-terminus promotes its transport inside mitochondria [189]. Therefore, adding an MTS to the enzyme, which specifically recognizes mutant mtDNA and introduces a DSB there, reduces the heteroplasmy level. Almost all instruments for the directed elimination of pathogenic mtDNA are based on the above principle, and these anti-genomic approaches are currently considered as the most promising future therapeutic methodologies for diseases caused by mtDNA mutations.

For the first time, Srivastava and Moraes applied this methodology to manipulate the heteroplasmy level [190]. They combined restriction endonuclease PstI with the COX8A mitochondrial localization signal (MLS). By using cybrid cells with mouse and rat mtDNA (having different numbers of PstI cleavage sites), it was demonstrated that the application of a mitochondrial restriction endonuclease causes a considerable heteroplasmic shift. Mt8993T>G mutation in the *ATP6* gene leads to the formation of an SmaI endonuclease recognition site. This fact was employed for the specific degradation of a mutant mtDNA using the SmaI enzyme containing the MLS [191]. The transient expression of modified SmaI in cybrid cells reduced the heteroplasmy level for mt8993T>G mutation and restored ATP production and mitochondrial membrane potential. Therefore, the above-mentioned works confirmed the effectiveness of mitochondrial restriction endonucleases for heteroplasmy level reduction for both scientific and potential therapeutic purposes.

However, the number of mutations forming unique recognition sites for restriction endonucleases limits the application of this approach. Despite the identification of novel mtDNA polymorphisms, only a small fraction of them could become a target for mitochondrial restriction endonucleases. In addition to that, the nuclear genome contains a large number of restriction sites; therefore, one should always consider the off-target effects there, which potentially lead to unnecessary mutations.

Mitochondrially targeted designer nucleases enabled at least partly overcoming this issue. Initially developed for the nuclear genome editing, the zinc finger nucleases (ZFNs) [192] and transcription activator-like effector nucleases (TALENs) [193] have been adapted for the elimination of pathogenic mtDNA variants. Such nucleases consist of three parts (the MLS, the DNA-binding domain, the nuclease catalytic domain) fused in one protein [194,195]. As for mitochondrial restriction endonucleases, the MLS comprised a peptide of a protein naturally imported into mitochondria [196]. A nuclease domain was taken from FokI nuclease, which produces DNA double-stranded breaks (DSBs) [197]. The binding specificity of engineered nucleases with pathogenic mtDNA molecules was promoted by a DNA-binding domain from a bacterial transcription activator-like effector (TALE) protein in case of mitochondrially targeted TALE nucleases (mitoTALENs) [194] or by zinc finger DNA-binding domain for mitochondrially targeted zinc finger-nucleases (mtZFNs) [195].

Transcription factors often contain zinc finger type DNA-binding domains [198], which, in turn, include the nuclear localization signal (NLS) [199]. For the effective mtZFN import to mitochondria and the reduction of off-target effects in the nuclear DNA, NLS should be eliminated [200]. Importantly, for mtZFNs and mtTALENs, one could construct a binding domain for virtually any DNA sequence [201], considerably expanding the spectrum of targetable mtDNA pathogenic mutations. MtZFNs and mtTALENs were successfully utilized to shift the heteroplasmy level for both point mutations and deletions of mtDNA to restore the affected mitochondrial functions [155,194,195,202,203,204,205]. Disadvantages of mtZFNs and mtTALENs include complex and laborious process of DNA-binding domain construction, which are associated with the repetitive nature of these domains for mtTALENs and the complicated screening process for specific mtZFNs.

All of the above-described nucleases are artificially made proteins; therefore, the recognition and binding of a target DNA sequence is based on DNA–protein interactions. Clustered Regularly Interspaced Short Palindromic Repeats(CRISPR) and CRISPR-associated protein 9 (Cas9)—CRISPR/Cas9 is a novel and popular technology for nuclear genome editing that employs a single-guide RNA (sgRNA)-mediated approach to target DNA sequences [206]. Target DNA recognition proceeds based on DNA–RNA binding, which makes it more specific compared to other methods. Additionally, CRISPR/Cas9 is more effective in genome editing and more flexible due to a simple procedure of the sgRNA customization [207]. Therefore, many groups including ours are working on the optimization of this system for mutant mtDNA elimination and mitochondrial genome editing. However, still it is far from an unambiguous confirmation for CRISPR/Cas9 effectiveness in mitochondria. Moreover, some researchers question even the theoretical possibility of its effective use for pathogenic mtDNA elimination [171]. Analogous to anti-replicative RNAs, such skepticism arises due to the lack of information on the molecular mechanisms of RNA transport inside mitochondria, speculating that the whole concept of mitochondrial CRISPR/Cas9 is controversial.

#### 4.2.3. MtDNA Base-Editing

MtDNA editing is an exciting concept because it is essential to correct pathogenic mtDNA mutations to treat mitochondrial diseases and also to introduce mutations into mtDNA to acquire disease models and study fundamental processes of mtDNA function. The existing nuclear genome editing methodology is based on a modified CRISPR/Cas9 system [208,209]; therefore, such direction for mtDNA editing seems controversial. However, very recently, a protein-only system (DddA-derived cytosine base editor, DdCBE) has been introduced. It is based on bacterial toxin DddA with deaminase activity [210]. This enzyme enables deaminating cytosine (C) preceded by a thymine (T) residue in a double-stranded DNA molecule. Deamination promotes C conversion to uracil (U), which pairs with adenine (A) residue. As a result, during mtDNA replication, A would be inserted complementary to U, and CG would be converted to a UA pair. Consequently, through DNA repair mechanisms, U is substituted by T residue, resulting in CG transition into TA. To reduce the level of unspecific deamination and cellular toxicity, the DddA domain was split in two halves, which were both fused to the programmable DNA-binding TALE domains, analogous to mtTALENs. Upon the binding of these proteins with two adjacent mtDNA motifs, two halves of DddA could associate to form a functional cytidine deaminase. Using human cell cultures, Mok et al. demonstrated that DdCBE effectively and specifically converts CG pairs into TA pairs. It does not show considerable off-target effects on both the mitochondrial and the nuclear DNA [210]. It is a promising technology for mitochondrial disease treatment because the majority of pathogenic mtDNA mutations are T > C transitions. Additionally, such an approach might be utilized to study the mechanisms of mitochondrial diseases associated with mtDNA mutations and aging.

## 5. Conclusions

Mitochondrial diseases develop due to inherited or spontaneous mtDNA mutations. During the evolution, negative selection mechanisms were established to discard defective copies of mtDNA. These mechanisms reduce but not eliminate the risk of the manifestation of mitochondrial pathologies. Considering that, it is crucial to develop and modify mtDNA editing tools. At the same time, the understanding of natural mechanisms of mtDNA heteroplasmy level would facilitate the invention of new methodologies of mitochondrial disease prevention and treatment.
